# COVID-19 mRNA vaccines are effective at stopping a nosy virus

**DOI:** 10.1016/j.omtn.2025.102458

**Published:** 2025-01-30

**Authors:** Uxue Beloki, Laura Salaberry, Carla Ortuño-Moya, Angie Molina, Cristian Smerdou

**Affiliations:** 1 Division of DNA and RNA Medicine, Cima Universidad de Navarra, Av. Pío XII 55, 31008 Pamplona, Spain; 2 Instituto de Investigación Sanitaria de Navarra (IdISNA) and CCUN, Pamplona, Spain; 3 Nanogrow Biotech, Montevideo, Uruguay

## Main text

The manuscript by Fricke et al. shows that mRNA vaccines against SARS-CoV-2 are able to confer protection against fatal disease even in the absence of neutralizing antibodies, which suggests that cellular responses could compensate for suboptimal antibody levels.^1^ Interestingly, they also showed that mRNA vaccines encoding for the spike protein of SARS-CoV-2, either N1mΨ modified or not, induced neutralizing immunoglobulin (Ig)Gs in the nasal conchae of mice, which were critical for achieving effective viral clearance in the upper respiratory tract, limiting disease severity, and likely helping prevent virus transmission, although this last possibility was not tested in this work.

**Figure 1:**
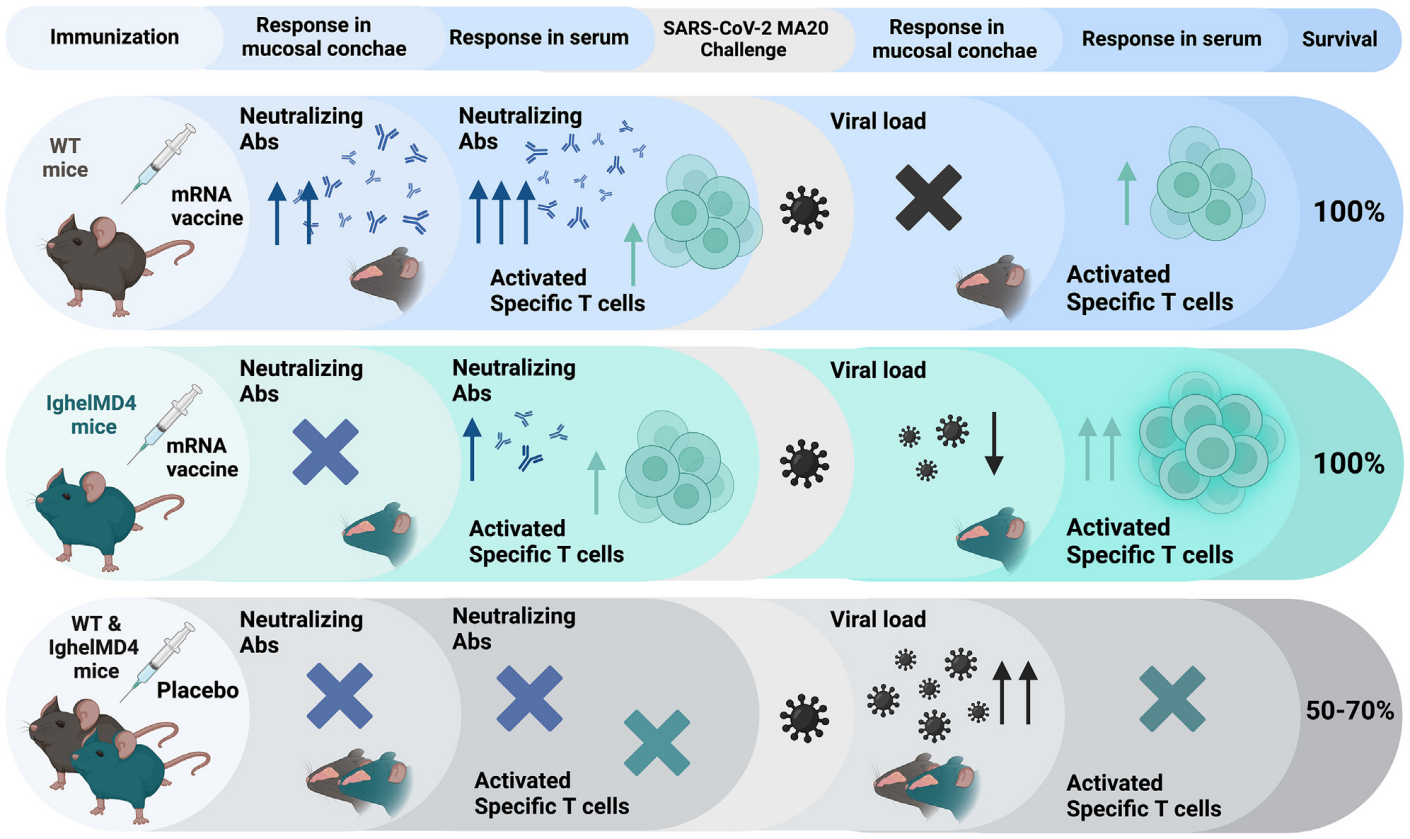
Vaccination of WT and IghelMD4 mice with COVID-19 mRNA vaccines A summary of the humoral and cellular responses induced by vaccination as well as the level of protection from lethal disease after challenge with a mouse-adapted SARS-CoV-2 (MA20) is shown. Abs, antibodies; specific T cells, SARS-CoV-2 spike-specific T cells. This figure was created with Biorender.com.

Despite the fact that the approved COVID-19 vaccines have been shown to protect from severe disease by inducing neutralizing antibodies, less information is available on the role of cellular immune responses against viral infection. This is an important issue since it could compromise the efficacy of vaccines in patients with humoral immunodeficiencies or undergoing immunosuppression treatments. In this regard, a recent study has shown that patients with multiple sclerosis receiving anti-CD20 antibody monotherapy were able to develop antiviral CD4^+^ and CD8^+^ T cell responses after vaccination with COVID-19 mRNA vaccines despite having suboptimal antibody responses.^2^ Although the degree of protection against severe COVID-19 disease in those patients is unknown, preclinical studies in mouse models lacking antibodies have shown that the immunity conferred by viral infection or mRNA vaccination can protect against SARS-CoV-2 challenge independently of antibodies.^3^ However, a frequent limitation of the mice models employed to assess the role of neutralizing antibodies is that they usually completely lack B lymphocytes or antibody production, which does not reflect the more variable situation in humans. For that reason, Fricke et al. decided to use in their study a mouse model with limited humoral responses (IghelMD4), which could better recapitulate the situation in human vaccinees having suboptimal humoral responses.^1^^,^^4^ When vaccinated with COVID-19 mRNA vaccines, these mice exhibited a significantly reduced capacity to generate circulating antibodies compared to wild-type (WT) littermates. Vaccinated IghelMD4 mice showed some degree of disease progression after being challenged with a mouse-adapted strain of SARS-CoV-2 (MA20), which was not observed in vaccinated WT mice. However, both strains of vaccinated mice were fully protected from lethal disease, in contrast to sham-vaccinated mice, which showed a 50%–70% survival (Figure 1). This suggests that virus-specific systemic antibodies induced by mRNA vaccination can attenuate disease progression, such as weight loss, but are not required to prevent lethal outcomes. To address the mechanisms of protection in the absence of antibodies, the authors analyzed in detail T cell populations in lungs and spleens of vaccinated mice, observing that both WT and IghelMD4 mice developed comparable virus-specific cellular responses after mRNA vaccination, with a pronounced activation of CD8^+^ T cells in the lungs showing an increase of activation markers, such as CD69, PD-1, and CXCR3. In addition, their analyses revealed that functional T cell responses, including interferon (IFN)-γ and granzyme B production, were comparable between WT and IghelMD4 mice, suggesting that T cells could play a crucial role in providing protection. Interestingly, upon virus challenge, IghelMD4 mice showed higher T cell activation than WT mice, which could represent a compensatory mechanism to protect against fatal disease in animals with low levels of neutralizing antibodies.

The induction of mucosal immunity has been proposed as being important to prevent infection and transmission of respiratory pathogens, including SARS-CoV-2.^5^ This aspect was also studied in the current work, observing that while neutralizing antibodies were completely absent in the nasal conchae of vaccinated IghelMD4 mice, WT mice showed antiviral IgGs at that site at least up to day 12 post-vaccination. In accordance with these data, IghelMD4 mice failed to control nasal viral loads, suggesting that mucosal IgG responses are essential for an effective clearance of SARS-CoV-2 virus from the upper respiratory tract, particularly during the early stages of infection, and may play a key role in limiting disease spread. It is important to note that T cell responses within the nasal mucosa were not evaluated in this study but could also potentially contribute to viral clearance. Given that the current study examined the immune responses only during 2 weeks post-infection, future research evaluating the long-term efficacy of mRNA vaccination would be of interest. Additionally, exploring alternative administration routes, like intranasal delivery, could help improve long-term immunity and boost mucosal protection.

In summary, this article highlights the complementary mechanisms of humoral and cellular immunity induced by mRNA vaccines. The results suggest that while neutralizing antibodies are crucial for effective viral control, T cell responses may offer an additional layer of protection when humoral responses are compromised. A deeper evaluation of how increased CD8^+^ T cell activation may affect long-term immunity, as well as a more in-depth study of the mechanisms by which T cells contribute to protection against severe COVID-19 disease, would be of interest. Overall, the findings of this work are particularly relevant for designing vaccination strategies for immunocompromised individuals or those with limited humoral responses. They also highlight the importance of developing intranasal or inhaled vaccine strategies to enhance mucosal immunity to better prevent viral transmission. Future research could help in developing more effective vaccines not only for SARS-CoV-2 but also for other respiratory pathogens.

## Acknowledgments

This work is funded by the following grants to C.S.: project PI23/00565 funded by Instituto de Salud Carlos III (ISCIII) and co-funded by the European Union, Gobierno de Navarra, Departamento de Salud (GN2022/21), and Fundación Intheos (Spain).

## Declaration of interests

L.S. is currently an employee of Nanogrow Biotech.

## References

[1] Fricke C., Ulrich L., Kochmann J., Gergen J., Kovacikova K., Roth N., Beer J., Schnepf D., Mettenleiter T.C., Rauch S. (2024). mRNA vaccine-induced IgG mediates nasal SARS-CoV-2 clearance in mice. Mol. Ther. Nucleic Acids.

[2] Apostolidis S.A., Kakara M., Painter M.M., Goel R.R., Mathew D., Lenzi K., Rezk A., Patterson K.R., Espinoza D.A., Kadri J.C. (2021). Cellular and humoral immune responses following SARS-CoV-2 mRNA vaccination in patients with multiple sclerosis on anti-CD20 therapy. Nat. Med..

[3] Fumagalli V., Ravà M., Marotta D., Di Lucia P., Bono E.B., Giustini L., De Leo F., Casalgrandi M., Monteleone E., Mouro V. (2024). Antibody-independent protection against heterologous SARS-CoV-2 challenge conferred by prior infection or vaccination. Nat. Immunol..

[4] Mason D.Y., Jones M., Goodnow C.C. (1992). Development and follicular localization of tolerant B lymphocytes in lysozyme/anti-lysozyme IgM/IgD transgenic mice. Int. Immunol..

[5] Cao K.T., Cobos-Uribe C., Knight N., Jonnalagadda R., Robinette C., Jaspers I., Rebuli M.E. (2023). SARS-CoV-2 mRNA vaccination induces an intranasal mucosal response characterized by neutralizing antibodies. J. Allergy Clin. Immunol. Glob..

